# Demographic forecast modelling using SSA-XGBoost for smart population management based on multi-sources data

**DOI:** 10.1371/journal.pone.0320298

**Published:** 2025-06-25

**Authors:** Jin Wang, Shihan Ma, Qing Lv, Qiang Li

**Affiliations:** 1 College of Engineering of Hebei Normal University, Shijiazhuang, China; 2 Hebei Provincial Key Laboratory of Information Fusion and Intelligent Control, Shijiazhuang, China; University 20 Aout 1955 skikda, Algeria, ALGERIA

## Abstract

Population prediction could provide effective data support for social and economic planning and decision-making, especially for the sub-national population forecasting accurately. In addition to realizing efficient smart population management, this research focuses primarily on the combination model for forecasting demographic data based on machine learning. As to the higher error of population forecasts due to high population density and mobility, a dynamic monitoring method based on mobile communication big data such as mobile phone signals is proposed, combined with more structurally stable traditional statistical data, it forms a multi-source dataset that possesses both accuracy and real-time characteristics. In the study, the Extreme Gradient Boosting tree (XGBoost) model is used to identify the base model to create a reliable predictive model for population dynamic monitoring. The sparrow search algorithm (SSA) is investigated to obtain more reasonable parameters of XGBoost to improve forecast accuracy. The combination model is verified based on the data of the 6th and 7th national population census and mobile phone signal data in Hebei Province, obtained the predicted data for mortality and migration, categorized by age and gender, for the following year. Subsequently, the research compared the performance of different metaheuristic algorithms and various gradient-boosting machine-learning models on the dataset. The SSA-XGBoost model demonstrates a better prediction performance in the demographic data forecast with better R^2^ 0.9984 and a lower mean absolute error of 0.0002 and a mean squared error of 6.9184. The results of the comparative experiments and cross-validation show that the proposed predictive model can effectively forecast the demographic data for sub-national regions to realize smart population management.

## Introduction

Rapid urbanization has led to a sharp increase in urban population density, complexity, and mobility. To address the challenges of rapid urbanization on high-quality urban governance and industrial development, generating accurate and reliable projections of population size, structure, and mobility is essential for decision-making, strategic planning, and meeting the requirements of diverse services and infrastructure, such as formulating public policy [1], allocating healthcare and educational resources [2,3], building smart city [4,5], and energy planning and management [6–8]. The demographic situations can be found and collected from sub-national areas, where provinces, cities, and Statistical Area Level 2 (SA2) [9,10] are examples of sub-national areas. Nevertheless, prediction methods based on these sub-national data as mentioned earlier are susceptible to generating highly inaccurate numbers. This is primarily attributed to the following factors: firstly, exponentially growing intra-regional population movement, migration, and social integration result in high complexity and error of population dynamics and forecast [11]; secondly, the development of populations shows complex non-linear and multi-dimensional features. Traditional forecasting methods, like time series analysis and demographic models [12–14], are often constrained by linear assumptions and limited dimensionality when predicting mortality, fertility, and migration using single-source data. In light of the rapid development of big data and digital technologies, Wilson, Grossman, and their colleagues contend that ensemble forecasting and machine learning algorithms present substantial research opportunities in the area of population prediction [15]. Meanwhile, the integration of mobile communication big data, such as mobile phone signaling, geographic location, satellite remote sensing, and social media data, into population prediction analysis offers robust data support for diversified and real-time forecasting. It also maximizes the strengths of machine learning and ensemble prediction models in handling complex, multi-dimensional, and large-scale data. Therefore, dynamic monitoring of population data by multi-resources is an important way to improve the accuracy of population forecasts.

This paper is expected to propose optimization solutions that improve the real-time accuracy of urban population prediction from two perspectives: data from multiple sources and ensemble models based on machine learning. Current studies on population prediction, both in domestic and international contexts, mainly depend on census data and traditional sample survey data [16]. Conducting censuses and surveys is costly in terms of human and material resources, involves multiple intermediary steps, and is susceptible to human error. Moreover, the extended time intervals between censuses limit the ability to perform fine-grained temporal forecasting. Variations in administrative regions also result in inconsistent survey methods, complicating the acquisition of statistical data for specific areas of interest. While acknowledging the limitations of traditional statistical methods, this research does not fully transition to the use of LBS data(including mobile phone signaling data, heatmaps, remote sensing data, etc.) as is common among many researchers [17,18]. The paper establishes a baseline using traditional statistical methods, such as population census and sampling surveys, and integrates real-time correction data from mobile communication platforms like Baidu Maps and mobile phone signaling, this multi-source data framework is used for dynamic population prediction. Research on population prediction models involves both the refinement of conventional methods, including linear regression [19], ARIMA [20], and Logistic models [21], and the exploration of new approaches using multiple intelligent algorithms and machine learning models for ensemble forecasting. The No Free Lunch Theorem [22] suggests that ensemble forecasting with multiple algorithms can yield better overall results. However, incorporating an excessive number of algorithms can greatly increase model complexity, resulting in higher overfitting, reduced real-time performance, and inefficient use of computational resources. This research develops an ensemble prediction model that includes the Salp Swarm Algorithm (SSA) for its superior parameter optimization and the Extreme Gradient Boosting (XGBoost) model as a robust baseline based on distributed gradient boosting.

The purpose of this research is to develop an ensemble prediction model combining meta-learning and machine-learning techniques, leveraging multi-source population data to forecast and analyze the population structure, birth and death rates, and migration patterns within a region. Specifically, the paper has the following two key research aims:

To build and curate a multi-source population dataset comprising traditional statistical sources such as population censuses and sampling surveys, along with mobile communication data from mobile phone signalling and Baidu Maps.

To establish a streamlined ensemble prediction model capable of automatically optimizing the hyperparameters of machine learning models, and to validate the model’s effectiveness in regional population forecasting through comparative analysis.

In this study, the researchers gathered population census data from 2010 and 2020, along with multiple rounds of sampling survey data between 2010 and 2020, and mobile phone signaling data from 2019 to 2020 for Hebei Province. By partitioning the dataset into training and validation sets, they obtained predicted population data for Hebei Province in 2020 classified by age and gender which is presented in section Data and methodology. This section also explains the base models, the combination methods, and the overall experimental design used in this study. The Result and analysis section presents population status and structure. The Discussion Section compares the performance of SSA-XGBoost model with other models.

## Literature review

Using communication data for population dynamic monitoring is a feasible method, which has become a hot spot in recent years [23–28]. For example, Calabrese et al have used mobile communication data to conduct real-time monitoring of the population of Rome [29]; Naaman et al. conducted a study on the daily behaviours of the urban population based on Twitter check-in data [30]. Based on mobile positioning data rarely, Martin Sveda et al [31] provide an appropriate method to transform data from the mobile network into target spatial units, ensuring the precision and accuracy of the results for population estimates. In addition, Yongping Zhang et al [32] utilize mobile phone data as a data source to investigate the working and residential segregation of migrants in Longgang City, China. Fabio Ricciato, et al [33] proposed an approach to the estimation of present population density from mobile network operator data collected by Mobile Network Operators (MNO). The operation difficulty of population statistics using mobile communication big data costs less manpower and material resources, so it can achieve high-frequency monitoring. Moreover, mobile data contains multiple dimensional attributes, including temporal and spatial information, user characteristics, and flow rules. However, multi-resource data exists the differences in multiple semantic natures, multiple-scale features, and storage formats, and there are differences in data models and storage structures.

Computational intelligence and machine learning methods have been very promising in the field of prediction. Recurrent neural networks [34] based on improved architectures such as Long Short-Term Memory (LSTM) and Gated Recurrent Units (GRU) exhibit strong time series prediction capabilities in scenarios like climate science [35], traffic flow [36] and emergency evacuation [37]. While these improved structures, such as LSTM and GRU, address some of the shortcomings of RNNs in terms of gradient issues and long-term dependency relationships to a certain extent, the fact remains that RNNs require multi-step backpropagation through time (BPTT), leading to longer training processes and higher consumption of computational resources. Given these challenges, machine learning algorithms based on gradient boosting decision trees (GBDT), like Adaboost [38], XGBoost [39], LightGBM [40], and CatBoost [41], have gradually come into research focus. Combining the fine-grained control over large-scale data and the generalization ability for prediction targets, the XGBoost model has become one of the preferred choices for many researchers. The XGBoost model is considered to be very flexible, and it can adapt to a variety of different problems. However, this also means that the hyperparameters have to be tuned for each of these specific tasks. Selecting the optimal values for hyperparameters through optimization can be regarded as a non-deterministic polynomial (NP) problem [42]. Metaheuristic optimizations, which use random operators, trial-and-error processes, and random scanning of the problem-solving space to generate efficient solutions to optimization problems [43], play a crucial role while dealing with NP-hard challenges that are commonly faced in parameter optimization, regression analysis, cluster analysis, etc.

There is limited research on stacking the XGBoost model with other metaheuristic algorithms for population prediction analysis and comparing the performance of the stacked model with other standalone machine learning models. Practitioners not only need to decide which individual models to integrate with XGBoost but also need to determine the method of integration to allocate weights among the algorithms. Relatively novel optimization algorithms like crayfish optimization algorithm [44], reptile search algorithm [45], red fox optimizer [46], sparrow search algorithm (SSA) [47], particle swarm optimization (PSO) [48], and other swarm algorithms have been utilized for obtaining more reasonable parameter settings to improve the predictive performance of the model. Mohamed Salb et al. [49] have designed an innovative solution that combines convolutional neural networks (CNNs) for feature extraction and the XGBoost model for intrusion detection, by customizing the reptile search algorithm for hyperparameter optimization, the methodology provides a resilient defence against emerging threats in IoT security. Tamara Zivkovic et al. [50] suggested a modified variant of the reptile search optimization algorithm named HARSA to carry out the calibrating of the XGBoost hyperparameters, by comparing with other metaheuristic algorithms, the proposed scheme has been shown to have superior classification accuracy. Mihailo Todorovic et al. [51] compared the performance of six metaheuristic algorithms used for tuning the XGBoost algorithm in their study. The results showed that models with hyperparameter optimization outperformed the benchmark models in financial data prediction. Nguyen Thi Thuy Linh et al. [52] evaluated the performance of the hybrid genetic algorithm (GA) optimization method and XGB mode land K-nearest neighbour. validated on the test dataset, using the genetic algorithm as an optimizer for determining the best parameters in the XGB model increases efficiency in this study. Luka Jovanovic et al. [53] tested eight metaheuristics algorithms for XGBoost optimization to achieve a superior level of performance in estimating the relative importance of each pollutant level and meteorological parameter for the prediction of benzene concentrations. Among these algorithms, the SSA is a population-based optimization algorithm that was proposed based on foraging and anti-predatory behaviours of sparrow populations and built upon existing population intelligence algorithms, such as GWO, GA, PSO, etc. It presents certain advantages in terms of stability, convergence accuracy, and velocity.

As discussed in the related literature, the combination of metaheuristic algorithms and machine learning models has been proven to improve model accuracy across various fields. XGBoost algorithm has high precision, strong flexibility, and can prevent data over-fitting, but this algorithm has high time and space complexity. The metaheuristic algorithm SSA, on the other hand, can further enhance the predictive performance of the XGBoost model through hyperparameter optimization. Therefore, a global optimization method based on SSA is proposed in this paper to identify the improved XGBoost model to realize population dynamic monitoring.

### Data and methodology

**Fig 1:**
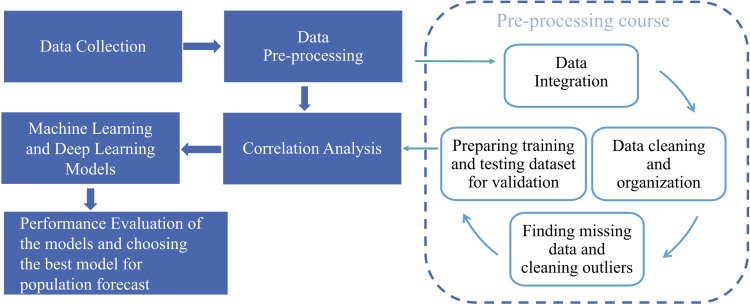
Methodology adopted for this study.

The methodology employed in this investigation is illustrated in Fig 1. The initial phase of the study started with data collection. After a data pre-processing stage, the acquired data is ready for modelling. Data pre-processing is a 4-stage process involving the following steps:

- Data Integration.
- Data cleaning and organization.
- Finding missing data and cleaning outliers.
- Generating data sets for training, testing, and validation.

The data pre-processing is then followed by correlation analysis to find out the correlation between input and output variables. The machine learning models will be proposed for demographic data. The model performance evaluation is then carried out using various metrics.

### Data collection

The following data are used in this study:

(1) National census data: These data were obtained from the 6th and 7th censuses, which could reflect the basic population situation, the change in family structure, the improvement of education level, and the population distribution differences among regions. The statistics from 2010 to 2020 cover the entire year, while the statistics for 2021 cover the first to the twelve months. The national census data can be obtained from the National Bureau of Statistics.
(2) Mobile information data: The main data source is mobile phone communication network signalling data, including mobility control (MC) port data and mobility management entity (MME) port data. MC port data is the location data of 2G/3G mobile phones, whose signal is generated for an average of about 20 minutes during the day. MME port data is 4G mobile phone location data, with every 5 minutes to generate data. Use the voice call CDRS to exclude numbers that have not received a voice call in the past six months. The 13-digit numbers starting with 106 and 144 are excluded. The mobile information data could be obtained by purchasing desensitization data from correlative communication companies.

Before data analysis and processing, the following two points about the population indicator statistical methods and the migration population are cleared as follow:

(1) Population indicator statistical methods
  a Stable population: The presence of more than 10 hours in the region on the same day is considered a stable day, and the number of stable days in a year is more than 1/2, which is considered a stable population in the month.
  b Population dimension (gender, age, residence registration location): Stable population associated ID number, gender identification bit to distinguish male and female. Stabilize the population associated with the ID number, and calculate the age according to the birth date. Precipitation analysis was conducted on children aged 0–17, who regularly visited children’s hospitals, primary and secondary schools, playgrounds, and other locations.
(2) Migration population: The intra-provincial stability of the current year minus the number of the previous year is taken as the inter-provincial migration object. The intra-provincial stability of the previous year minus the one of the current years is taken as the inter-provincial migration object. Take the roaming place of users in Hebei as the stable place, and take the mobile number home place as the stable place for users in other provinces.

### Data pre-processing

Data pre-processing mainly involves dealing with null or missing values in the data which need to be removed before modeling and the outliers also need to be removed before using it in a model. The data was grouped to identify the missing values. The outliers were identified as per Inter Quantile Range (IQR) [54]


IQR=Q3−Q1 (1)

where Q1 is first quartile corresponds to 25%, Q1 is the third quartile corresponds to 75%. The range considered was $\left[\begin{array}{cc} {}*20cQ1-1.5*IQR & Q3+1.5*IQR \end{array}\right]$.

The outlier points are seen in the data either due to a faulty detection or maybe an exceptional event. To ensure the comparability of the prediction, feature scaling was implemented consistently. The training set is denoted by and $X=\left(x_{1},x_{2},\ldots x_{n}\right)$ represents the n-dimensional explanatory space and y is the dependent variable. The normalization is denoted as:


$$X_{i}^{\prime }=\frac{X_{i}-\min(x)}{\max\left(x\right)-\min\left(x\right)}$$ (2)

## Machine learning algorithms

In recent years, the field of data science has brought machine learning and artificial intelligence to the forefront, and numerous machine learning algorithms have either emerged or have gained popularity. In this paper, XGBoost is adapted to make predictions iteratively on the training dataset and avoid too many splits, reduce the complexity of the model, and prevent the model from overfitting.

### XGBoost model

XGBoost is an algorithm based on gradient-boosting decision tree (GBDT). Compared with GBDT, XGBoost uses Taylor expansion to optimize loss function, and regularization term to avoid model overfitting. The loss function is expanded to the second order, and a regularization term is added to control model complexity. The objective function is composed of two parts: the loss function and the regularization term. The loss function is defined as:


$$L(\phi )=\sum_{i=1}^{n}l(y_{i}^{\prime },y_{i})+\sum_{k}\Omega (f_{k})$$ (3)

where n is the number of training samples, l is the loss function for an individual sample. $y_{i}^{\prime }$ is the predicted value for the $i^{th}$ training sample. $y_{i}$ is the true value for the $i^{th}$ training sample. $f_{k}$ could be defined as follows:


$$\begin{array}{cc} {}*20cf_{k}(x)=wq(x), & w\in RT,q:Rd\to \{1,2,.,T\} \end{array}$$ (4)

where w is weight vector of leaf node, q(⬝) is the mapping between leaf nodes.

Then, the complexity Ω of a tree is expressed as follows:


$$\Omega \left(f_{k}\right)=\gamma T+\frac{1}{2}\lambda \sum {\mathrm{\backslash nolimits}}_{J=1}^{t}w_{j}^{2}$$ (5)

where γ and is the penalty coefficient, T is the number of leaf nodes. And λ is punishing the score of leaf nodes.

### SSA algorithm

Sparrow Search Algorithm (SSA) is a novel swarm intelligence optimization algorithm inspired by the foraging and anti-predation behaviour of sparrows. In the process of sparrow foraging, it is divided into discoverer (seeker) and joiner (follower). The discoverer is responsible for finding food and providing foraging areas and directions for the whole sparrow population, while the joiner uses the discovery to obtain food. In addition, sparrow populations make anti-predation when they know the danger. In SSA, discoverers with better fitness values will preferentially obtain food during the search process. During each iteration, the location update of the discoverer is described as follows:


$$x_{ij}^{i+1}=\left\{ \begin{array}{c} \begin{array}{cc} {}*20cx_{ij}^{t}*\exp\left(\frac{-i}{\alpha *iter_{\max}}\right), & R_{2}<ST \end{array}\\ \begin{array}{cc} {}*20cx_{ij}^{t}+QL, & \begin{array}{cc} {}*20cR_{2}\geq ST & \end{array} \end{array} \end{array}\right.$$ (6)

where t denotes the current number of iterations, and $iter_{\max}$ is a constant that denotes the utmost number of iterations. $X_{ij}^{t}$ represents the position information of the $i^{th}$ sparrow in dimension $j^{th}$. α∈(0,1] is a random number. $R_{2}$ and ST represent the safety value and warning value respectively. *Q* is a random number that follows a normal distribution. L represents a 1×d matrix in which each element in the matrix is 1.

The joiner obtains food from the seekers. The continuously updated location of the joiner is as follows:


$$x_{ij}^{i+1}=\left\{ \begin{array}{c} \begin{array}{cc} {}*20cQ\cdot \exp\left(\frac{X_{worst}^{t}-X_{ij}^{t}}{i^{2}}\right), & i>n/2 \end{array}\\ \begin{array}{cc} {}*20cX_{P}^{t+1}+\left|X_{ij}^{t}-X_{P}^{t+1}\right|\cdot a^{+}\cdot L, & otherwise \end{array} \end{array}\right.$$ (7)

where $X_{P}$ denote the producer’s optimal position; $X_{worst}$ is the sparrow population’s worst position. $a^{+}$ denotes a matrix assigning 1 or −1 randomly at each element, and its dimension is 1 × *d*. The $i^{th}$ scroungers are starving with low fitness when i>n/2. When the sparrow population detects danger, sparrows at the edges quickly move to a safer area. The middle sparrow of the flock will approach other sparrows at random. The sparrows update their positions according to the following formula:


$$x_{ij}^{i+1}=\left\{ \begin{array}{c} \begin{array}{cc} {}*20cx_{best}^{t}+\beta \cdot \left|x_{ij}^{t}-x_{best}^{t}\right|, & l_{i}>l_{g} \end{array}\\ \begin{array}{cc} {}*20cx_{ij}^{t}+n\cdot \left(\frac{x_{ij}^{t}-x_{worst}^{t}}{(l_{i}-l_{w})+\varepsilon }\right), & l_{i}=l_{g} \end{array} \end{array}\right.$$ (8)

where $X_{best}$ is the best position of a whole sparrow population; β is defined as the control parameter of step size. $l_{i}$ and $l_{g}$ are the fitness values of present, current global best, and worst, respectively. When $\;l_{i}>l_{g}$, it means the sparrow is located at the edge of the whole group. When $\;l_{i}=l_{g}$, the middle sparrows of the flock spotted the danger and had to move closer to other sparrows. n determines the sparrow’s movement direction. ε denotes a small constant.

**Algorithm 1** The framework of SSA.

Input:

G: the maximum iterations; PD: the number of producers; SD: the number of sparrows who perceive the danger; $R_{2}$: the alarm value; n: the number of sparrows

Initialize a population of n sparrows and define its relevant parameters.

Output: $X_{best},f_{g}$

1: while (t<G)

2: Rank the fitness values and find the current best individual and the current worst individual.

3: $R_{2}=rand(1)$

4: for i=1:PD

5: Using equation (6) update the sparrow’s location;

6: end for

7: for i=(PD+1):n

8: Using equation (7)update the sparrow’s location;

9: end for

10: for I=1:SD

11: Using equation (8) update the sparrow’s location;

12: end for

13: Get the current new location;

14: If the new location is better than before, update it;

15: t=t+1

16: end while

17:return$X_{best},f_{g}$

### SSA-based parameter optimization

In the proposed method, each sparrow represents a set of XGBoost parameters, and the positions represent the parameter values. The mean square error of cross-validation is the objective function. SSA is employed to identify parameter values that minimize the objective function. The goal is to find a location $x_{i}$ that minimizes the objective function f(x), i.e.,


$$x^{*}=\mathrm{\backslash arg}\min f(x)$$ (8)

where x denotes the position of each sparrow is denoted. With each iteration, each sparrow position updates by the subsequent formula:


$$x_{i}^{*}=x_{i}+\alpha \cdot step\_size\cdot (x_{best}-x_{i})+\varepsilon$$ (9)

where $x_{best}$ is the current best sparrow position, α is a learning rate parameter, step_size is the step size, and ε is a random perturbation term. According to (10), the fitness of each sparrow is calculated and the position of the current best sparrow is updated. If the fitness of a specific sparrow exceeds the current best one, then the sparrow’s position is updated by the best value. This process is repeated until the optimal solution remains unchanged within a specified number of iterations, or until a predetermined number of iterations is reached.

Ultimately, the optimal sparrow position is the optimal solution required by the model and the parameters are shown in Table 1.

**Table 1:** The main parameters in the model.

Parameter name	Description	Value range
max_depth	control the tree size and affect the model complexity	(3,10)
learning_rate	control the weight reduction of each step	(0.01, 0.3)
subsample	control the proportion of data samples in each iteration	(0.7, 1)
colsample_bytree	control the proportion of features at each iteration	(0.7, 1)

**Fig 2:**
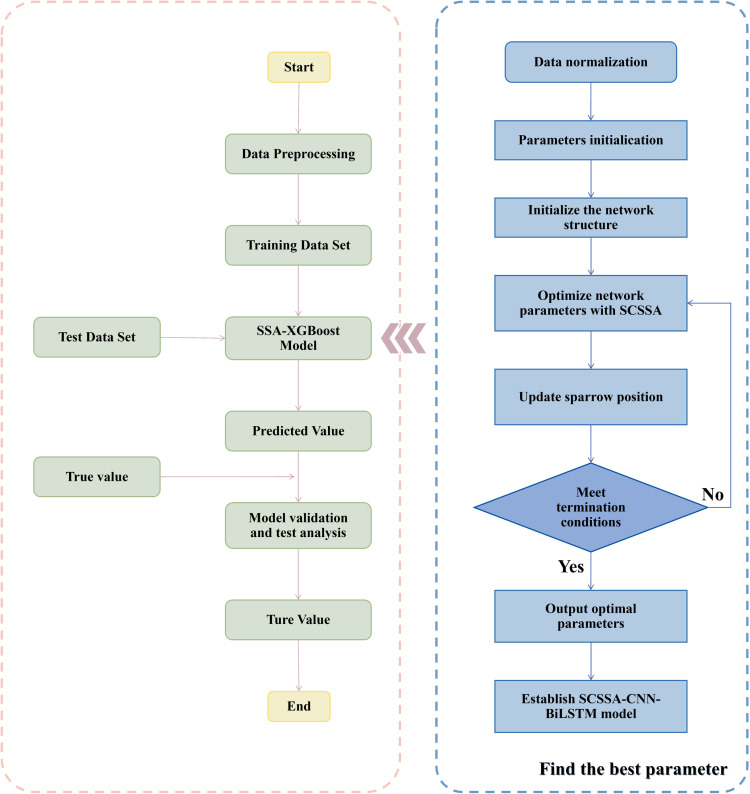
Flowchart of SSA-XGBoost model.

In the process of optimizing a population prediction system model based on SSA-XGBoost, each parameter is treated as a “sparrow” and the optimal parameter value is by simulating the sparrow’s foraging and anti-preying behaviour. The flowchart of the SSA-XGBoost model is shown in Fig 2.

### Evaluation indices

Evaluation is a critical stage in the implementation of any research project. Each model or procedure that is implemented must undergo an assessment using one or more metrics. The various model evaluation metrics used in the study are as follows:


$$R^{2}={\left[\frac{1}{n}\left(\frac{\sum {\mathrm{\backslash nolimits}}_{i=1}^{n}\left[\left(y_{i}-y_{m}\right)\left(x_{i}-x_{m}\right)\right]}{\sigma_{x}\sigma_{y}}\right)\right]}^{2}$$ (10)

$$MAE=\frac{\sum {\mathrm{\backslash nolimits}}_{i-1}^{n}\left|y_{i}-x_{i}\right|}{n}$$ (11)

$$MSE=\frac{\sum {\mathrm{\backslash nolimits}}_{i=1}^{n}{\left(y_{i}-x_{i}\right)}^{2}}{n}$$ (12)

where, n is the number of observations, $\sigma_{x}$ and $\sigma_{y}$ are the standard deviation of X and X respectively, $y_{m}$ and $x_{m}$ observed values respectively. $y_{i}$ is predicted value, $x_{i}$ is the actual value.

## Results and analysis

### Study area

In this paper, the dynamic management of population data in the Hebei Province of China is taken as an example due to its profound impact on the enhancement of regional comprehensive carrying capacity, economic development imbalance, and sustainable development. Compared with the data of the sixth population census in 2010, the number of separated households increased by 11,478,362 people, an increase of 138.34% in Hebei Province. The floating population increased by 8,657,908 people, an increase of 129.71%, with the inter-provincial floating population accounting for 20.58% and the provincial floating population accounting for 79.42%.

### Population status

#### The population’s natural growth rate is very close to zero, and population growth has substantially decreased.

**Fig 3:**
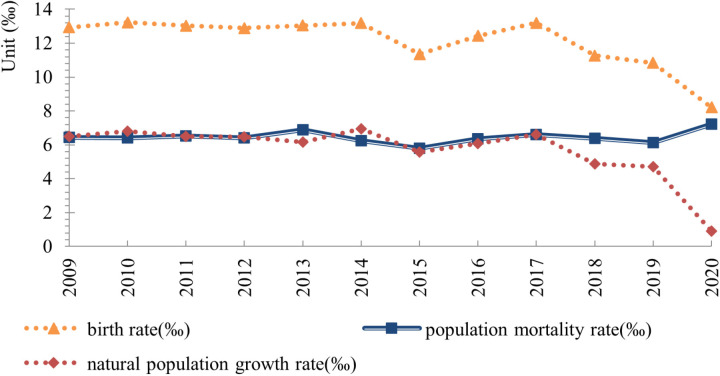
The natural change of population in Hebei Province from 2009 to 2020. (Data source: National Bureau of Statistics). Note: The population of 2000 and 2001 is the projected figure of the current population census, the population of other years is the projected data of the annual population sampling survey, and the population data of each region since 2005 is the standard of the permanent population.

The birth rate has fluctuated resulting in an accelerated decline in the population size of Hebei Province over the past decade. The birth rate experienced a brief surge in 2013, 2014, 2016, and 2017, following the implementation of the “two-child only” and “two-child universal” policies. Subsequently, it experienced a gradual decline. In 2020, it is anticipated to decrease to 8.2 per thousand. The mortality rate is 7.22 per thousand, with the rate fluctuating at a low level. In general, it has reached the stage of low birth rate, low mortality rate, and low natural growth rate, as illustrated in Fig 3.

#### Changes in the number of offspring, working-age population, and elderly.

**Fig 4:**
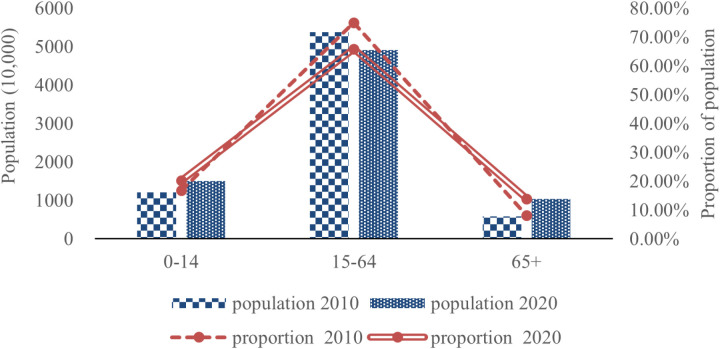
Changes in the number and proportion of children, working-age, and elderly in Hebei Province. (Data source: The sixth and seventh population censuses of Hebei Province).

In the past decade, the proportion of the working-age population decreased, while the number of infants and elders increased in Hebei province. The data from the seventh population census indicates that the number of children aged 0–14 years in Hebei Province reached 15.09 million in 2020, a 2.99 million increase from 2010. Additionally, the proportion of children aged 0–14 years rose from 16.83% in 2010 to 20.22%. The working-age population decreased from 53.84 million to 49.13 million, a decrease of 4.71 million. The proportion of the population aged 15–64 decreased from 74.93% to 65.86%. The number of geriatric individuals aged 65 and older increased from 5.92 million in 2010 to 10.39 million in 2020, a 4.47 million increase. Concurrently, the percentage of individuals aged 65 and older rose from 8.24% to 13.92%. The details are shown in Fig 4.

### Population structure analysis

**Fig 5:**
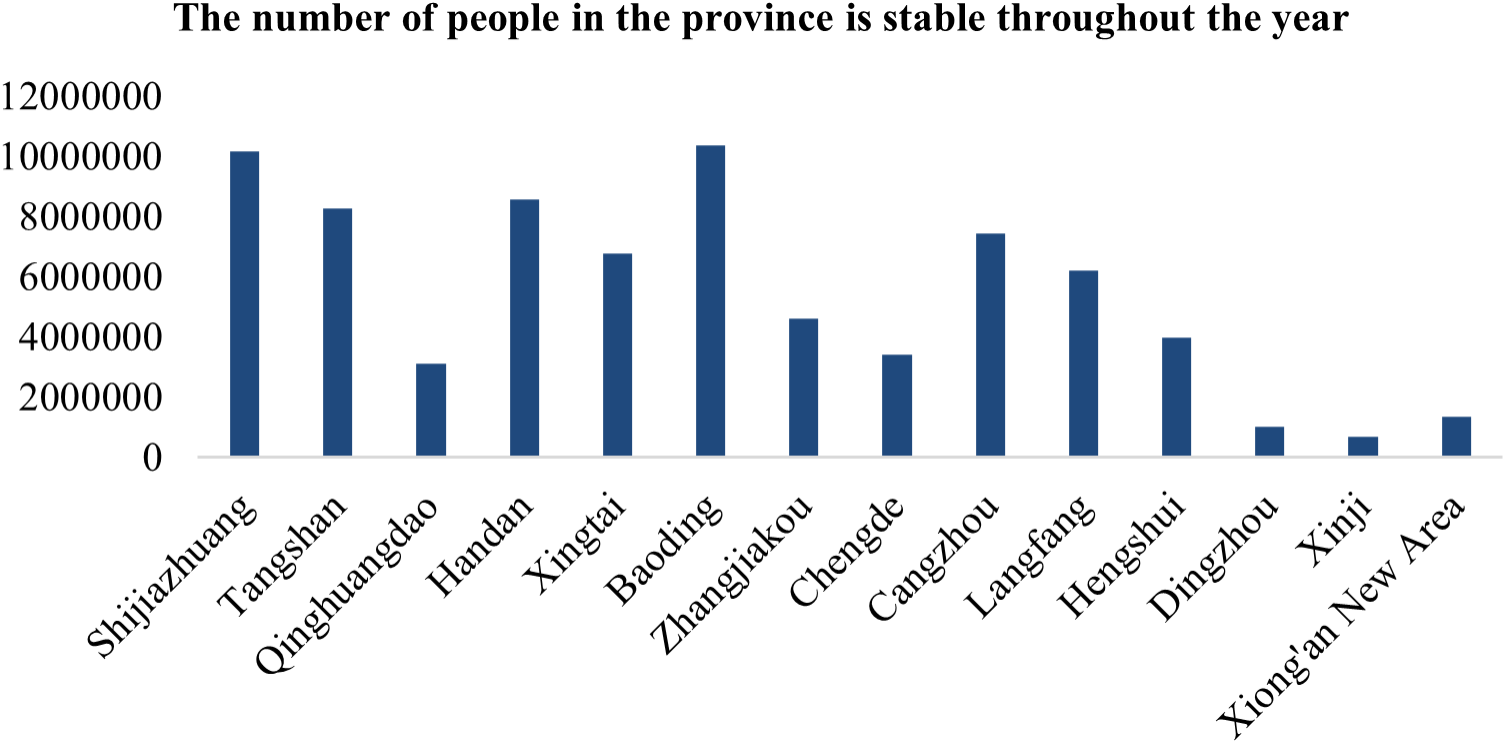
Stable population by region in Hebei Province in 2020.

According to the statistical data of mobile signaling in 2020 and 2021 (see Fig 5), the stable population of Hebei Province and prefecture-level cities in 2020 amounted to 75,996,000, and the stable population in 2021 amounted to 74,697,000, a year-on-year decrease of 1.71%. The top three cities with the largest stable population in 2020 were Baoding, Shijiazhuang, and Handan. In 2021, the top three cities with the largest stable population are Shijiazhuang, Baoding, and Tangshan, while the cities with the least stable population are Hengshui, Chengde and Qinhuangdao. It can be seen that the population size of cities in Hebei Province does not change much, and there is a positive correlation between population size and urban location, economic level and traffic conditions.

**Fig 6:**
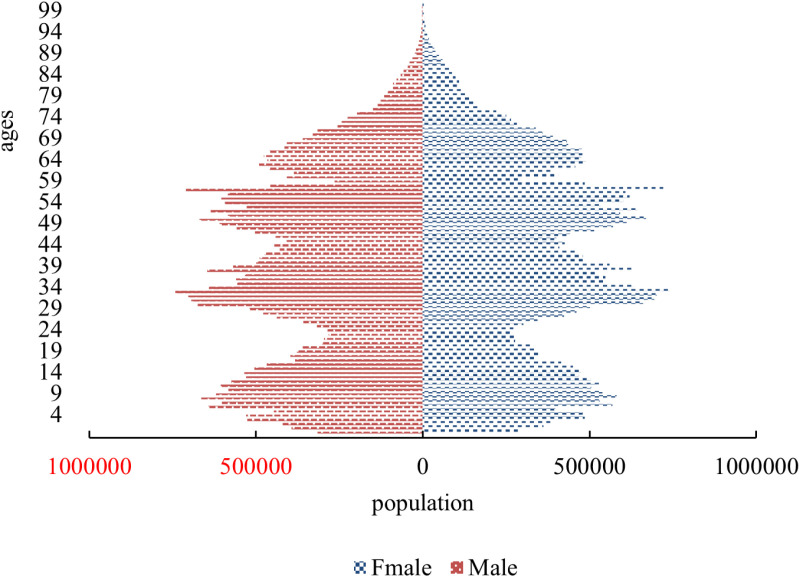
Population age pyramid of Hebei Province in 2020.

Fig 6 is the demographic structure of the population in Hebei Province in 2020, respectively. the population pyramid of Hebei Province is ageing, with a gradual decrease in the lower echelons. However, the working-age population continues to dominate the province, with a concentration of youthful and middle-aged individuals between the ages of 30 and 59.

As to the data, the population migration data comes from the 2020 Hebei Unicom mobile signalling data and Baidu VIP big data, and the GNP data of each region comes from the economic census data of the National Bureau of Statistics.

### Population prediction comparison

**Fig 7:**
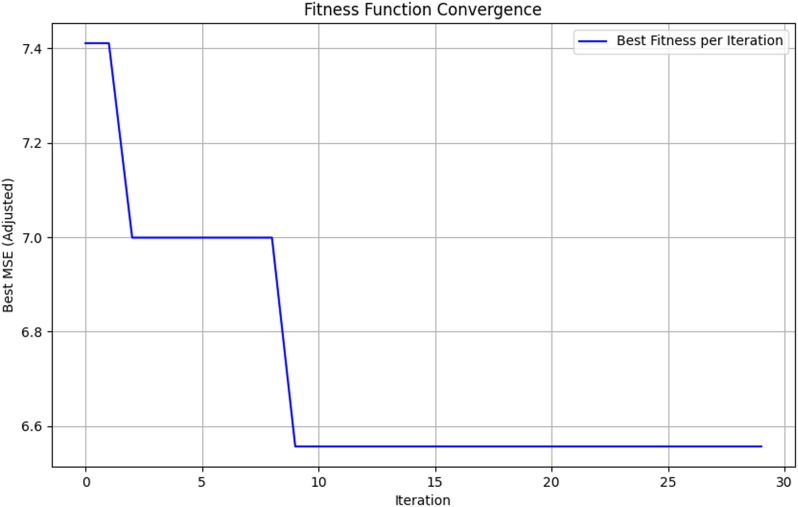
SSA fitness curve.

**Fig 8:**
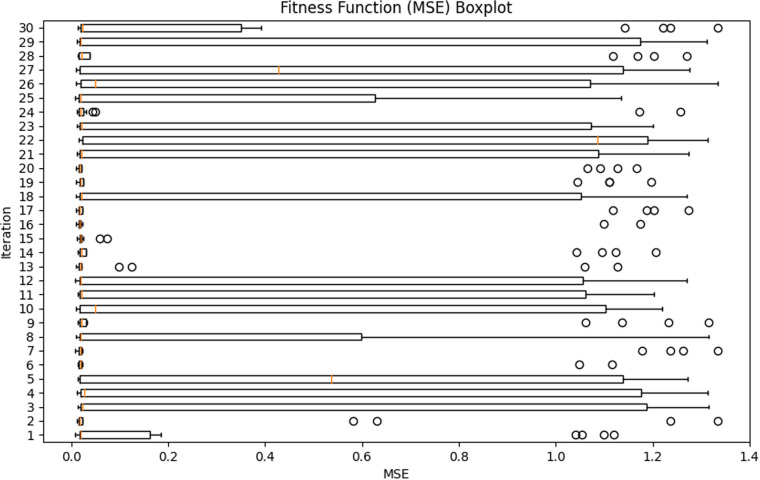
Box plots of the fitness function across independent runs.

In this section, the SSA algorithm is used to optimize the parameters of the XGBoost model to obtain reasonable parameter values. Twenty-five runs were conducted for the metaheuristic method, using a size of 10 solutions and a maximum of thirty rounds in each run (iterations = 10), which can be seen in Table 2. MSE has been utilized as an objective function that is required to be minimized throughout the conducted experiments. Figs 7 and 8 show the visualizations of the experimental outcomes in the form of the following graphs for both the fitness function: convergence graph and box plot. The optimal parameters and the fitness value are shown in Table 3.

**Table 2:** Parameter Values of the models.

Parameters	max_depth	learning_rate	subsample	colsample_bytree
Range of each Parameters	[3,10]	[0.01,0.3]	[0.7,1]	[0.7,1]
Optimum values	8	0.1900	0.7	0.8571
Best fitness	11635.3426			

**Table 3:** Experiment Settings.

Iterations	Solutions in the population	Independent runs	Solutions encoding
30	10	25	0.29373154 0.32396931 0.28318978 0.72868699

**Fig 9:**
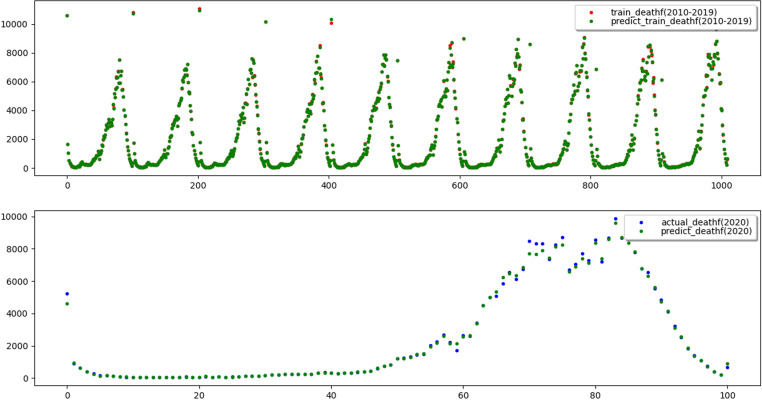
Results of training and prediction of death population for traditional XGBoost model.

**Fig 10:**
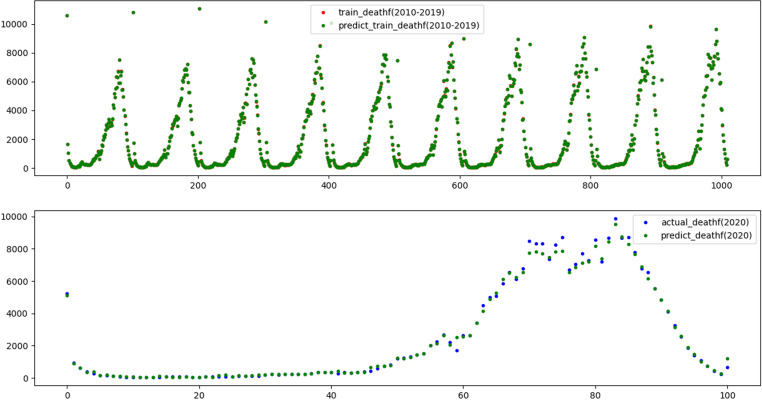
Results of training and prediction of death population for SSA-XGBoost model.

**Fig 11:**
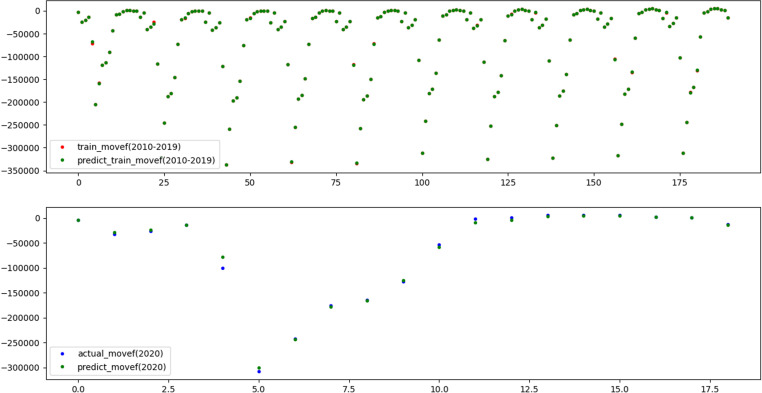
Results of training and prediction of migration data for traditional XGBoost model.

**Fig 12:**
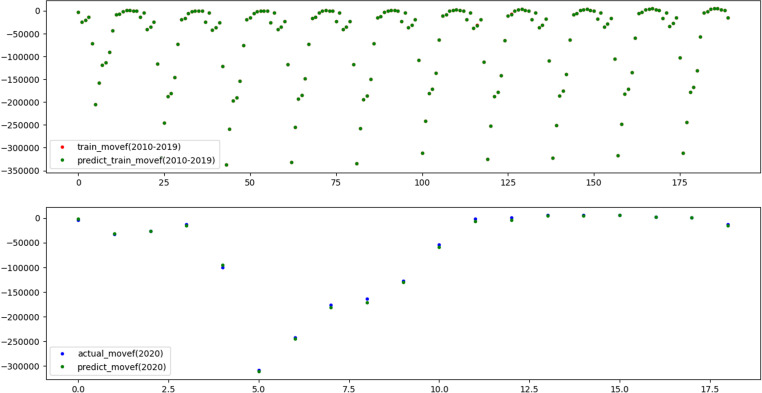
Results of training and prediction of migration data for SSA-XGBoost model.

To reflect the improvement of the prediction accuracy of the proposed method, the traditional XGBoost model and SSA-XGBoost model are adopted to predict mortality and mobility in Hebei Province. The results are shown in Figs 9–12, respectively.

Three evaluation indices were used to quantitatively evaluate the prediction effects of the two models, and the results are shown in Table 4, which shows that the adequacy of both models was determined to be satisfactory in terms of the $R^{2}$ values for the training and test data, as they were above 0.99.

**Table 4:** Statistics of the predictive performance indicators of the two models.

	Indices	XGBoost	SSA-XGBoost
Death	R^2^ (Test)	0.9946	0.9984
	R^2^ (Train)	0.9999	0.9998
	MAE	0.0017	0.0002
	MSE	11.5870	6.9184
Migration	R^2^ (Test)	0.9958	0.9979
	R^2^ (Train)	1.0000	1.0000
	MAE	0.0686	0.0005
	MSE	144.2836	0.4414

Nevertheless, $R^{2}$ value of 1 and 0.9999 on the training set indicates that XGBoost fully fits the training data, but it performs poorly on the test set. The results illustrate that SSA-XGBoost is preferable to traditional XGBoost in a variety of data indexes and efficiently prevents overfitting by incorporating regularization, and the prediction accuracy was significantly improved by parameter optimization.

**Fig 13:**
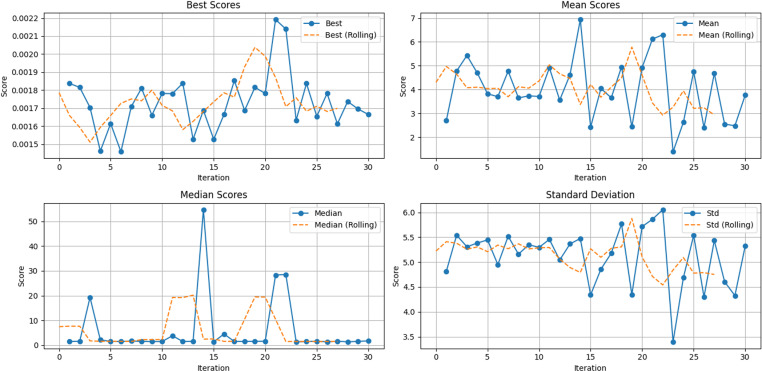
Performance metrics of cross-validation over iterations.

SSA-XGBoost performs best when the data set is death according to Table 4. To further verify the accuracy and stability of the model, SSA-XGBoost was cross-validated based on the death set, and the results are shown in Fig 13. The trend of Best Scores and Mean Scores shows a clear downward trend over the iterations, indicating that the model is continuously optimizing and finding better parameter combinations. Although the median Scores are not steady because of the outliers or missing values, the standard deviation continues to decrease over iteration. The rolling average line provides a smoother representation of this trend, further confirming the increase in stability.

## Discussion

### Comparisons with several baseline models

To better explore the prediction ability and universality of the SSA-XGBoost model, a variety of network models are used to forecast female deaths in 2020 and compare the predicted results. Considering that XGBoost is an optimized distribution gradient lift tree that belongs to machine learning, the models selected for comparison in this section are SSA-Adaboost and SSA-Catboost.

Three evaluation indices were used to quantitatively evaluate the prediction effects of the two models, and the specific values are shown in Table 5. All the evaluation indices indicated that the SSA-XGBoost model achieved the most accurate regression effect, and the prediction accuracy was significantly improved by parameter optimization.

**Table 5:** Statistics of the predictive performance indicators of three different models.

Indices	SSA-XGBoost	SSA-Adaboost	SSA-Catboost
R^2^	0.9984	0.908508	0.99488
MAE	0.0002	731.3905	129.4424
MSE	6.9184	709111.5	32387.68

**Fig 14:**
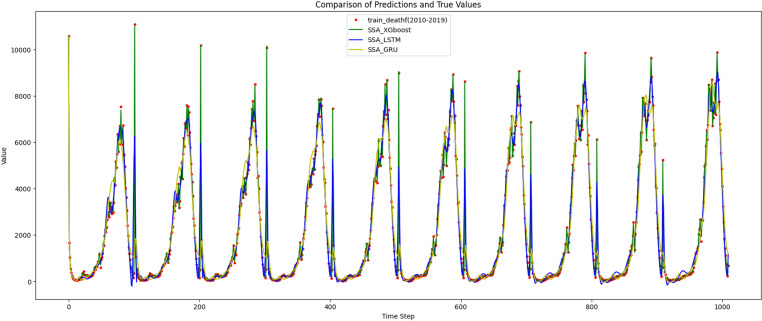
Comparison of prediction and true values for the training datasets.

**Fig 15:**
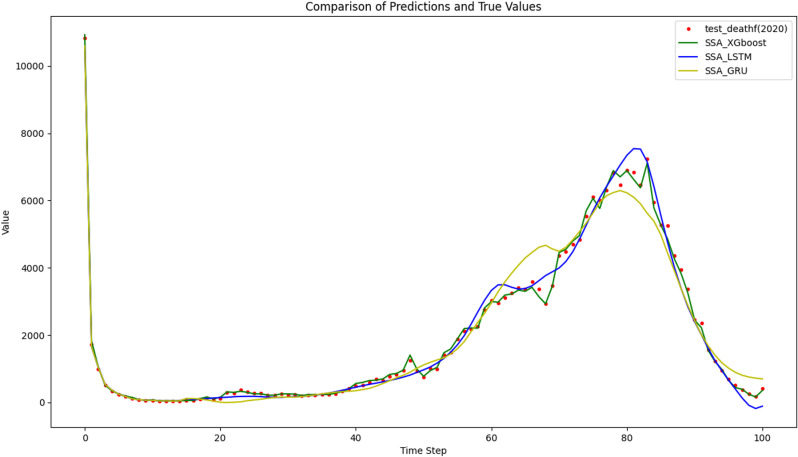
Comparison of prediction and true values for the test datasets.

As for deep learning^,^ RNNs like LSTM or GRU, are well known to handle time series well. The train(2010–2019) and test(2020) data set prediction results for the three models are shown in Figs 14 and 15.

The prediction curves of the LSTM and GRU models in Fig 15 are relatively smooth. LSTM performs well in capturing both short-term and long-term dependencies in the data. Its prediction curve closely follows the actual data, particularly at peaks and troughs. Similar to the LSTM, the GRU model is good at handling sequential data. Its predictions align well with the actual data, although there may be slight deviations during some significant changes in the data. The green line representing XGBoost is closely aligned with the red dots representing the actual data, indicating that it has accurately captured the trends and patterns in the data. As the four corresponding predictive performance indices shown in Table 6, the SSA-XGBoost model has the strongest prediction ability among the three models and the prediction error is relatively low.

**Table 6:** Statistics of the predictive performance indicators of the three models.

Indices	SSA-XGBoost	SSA-LSTM	SSA-GRU
R^2^ (test)	0.9984	0.9711	0.9539
R^2^ (train)	0.9998	0.9549	0.8913
MAE	0.0002	0.0004	0.0006
MSE	6.9184	9.4352	21.98922

### Comparison of different competitor algorithms

This subsection outlines the simulation results over the death data set with the XGBoost model optimized by SAA and other three recent competitor algorithms, including Crayfish, Reptile and Redfox. The pseudocode for each metaheuristic algorithm utilized is outlined in Algorithms 2–4.

**Algorithm 2** The framework of Crayfish.

1. Initialize the population of crayfish randomly.

2. Evaluate the fitness of each crayfish.

3. While stopping condition is not met:

   a. For each crayfish, determine its state (e.g., foraging, resting, or defending).

   b. Update the crayfish’s position based on attraction and repulsion forces:

      i. Attraction force: towards better solutions or prey.

      ii. Repulsion force: away from predators or danger zones.

   c. Foraging and defence behaviors:

      i. Simulate the crayfish searching for food while avoiding predators.

      ii. Update position accordingly.

   d. Evaluate the fitness of each crayfish’s new position.

   e. Update the global best solution if a better one is found.

4. Return the global best solution found.

**Algorithm 3** The framework of Reptile.

1. Initialize a population of reptiles randomly within the search space.

2. Evaluate the fitness of each reptile.

3. While stopping condition is not met:

   a. For each reptile, simulate movement using:

      i. Exploration: Move to a random direction (search for new solutions).

      ii. Exploitation: Move towards known better solutions (use past knowledge).

   b. Account for territorial behavior:

      If the reptile encounters others, simulate conflict or cooperation.

   c. Evaluate the fitness of each reptile’s new position.

   d. Update the global best position if necessary.

4. Return the best solution found.

**Algorithm 4** The framework of Red Fox.

1. Initialize a population of red foxes randomly within the search space.

2. Evaluate the fitness of each red fox.

3. While stopping condition is not met:

   a. Each red fox selects a strategy based on its current position:

      i. Search for food: move toward higher fitness (search for better solutions).

      ii. Escape predators: move to avoid worse solutions or stagnation.

      iii. Territorial behavior: defend a region or seek mates (exploit known good regions).

   b. Update the fox’s position based on its strategy.

   c. Evaluate the fitness of the updated position.

   d. Update the global best solution if a better one is found.

4. Return the best solution found.

Then, Table 7 exhibits the indices of XGBoost based on different optimal algorithms. The SSA-XGBoost model has the strongest prediction ability among four optimal algorithms and the indices are relatively low.

**Table 7:** Statistics of the predictive performance indicators of four different competitor algorithms.

Method	Number of runs	Best	Worst	Mean	Median	Std	Var
SSA-XGB	25	0.0031	11.7654	3.9569	1.2336	4.4900	20.1604
Crayfish-XGB	21	1.4928	16.1055	6.3965	5.0626	4.8960	23.9704
Reptile-XGB	20	0.1624	9.8134	2.8823	1.7353	3.0366	9.2208
Redfox-XGB	23	0.9376	22.1970	6.3955	5.0626	5.8736	34.4988

**Fig 16:**
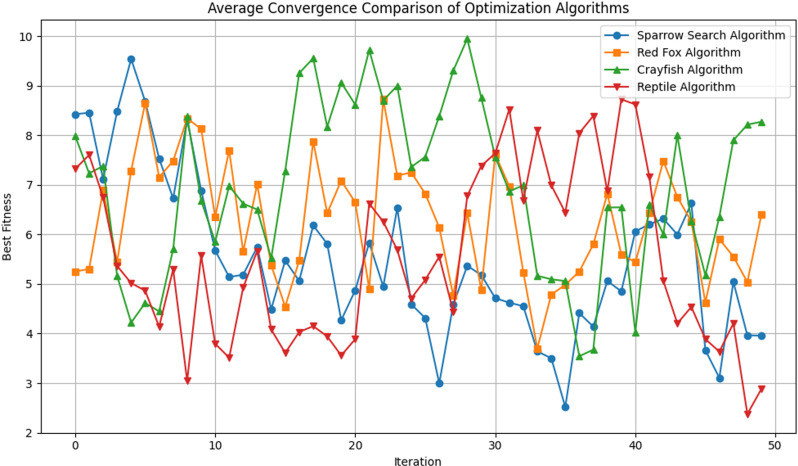
Visualized XGBoost simulations for all four metaheuristics regarding the convergence.

**Fig 17:**
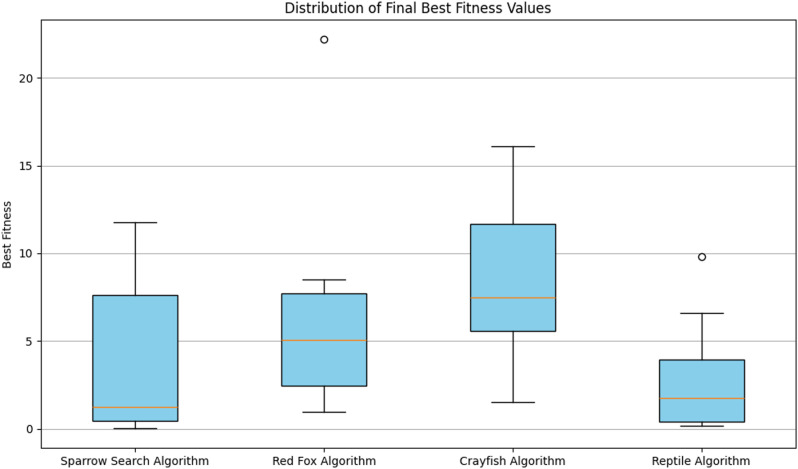
Visualized XGBoost simulations for all four metaheuristics regarding the box plot.

Figs 16 and 17 show the visualizations of the experimental outcome in the form of the following graphs.

## Conclusions

In this paper, a combined prediction model named SSA-XGBoost is proposed with the use of a sparrow search algorithm to optimize the parameters of the XGBoost model. Based on the 7th national population census of Hebei Province and the mobile communication data, a prediction experiment was conducted for a comparative analysis. Compared with the traditional XGBoost model, different metaheuristic algorithms (Crayfish, Reptile, and Redfox) and other models including deep learning and machine-learning(LSTM, GRU, CatBoost, and AdaBoost) through a variety of comparison graphs and error evaluation indicators, the following conclusions can be drawn:

(1) Compared with the traditional XGBoost model, the SSA algorithm was used to obtain more reasonable parameters to fit the actual development curve of the population, which greatly improves the ability of the model to predict time series. Concerning the population prediction, the SSA-XGBoost model is far better than the other models in terms of both sequence fit and performance evaluation indicators. This shows that the SSA and XGBoost combination model has a better prediction performance than their single models.
(2) The SSA-XGBoost model proposed in this study performs better in population prediction. Compared to other machine-learning models(CatBoost and AdaBoost), the XGBoost model optimized by SSA represents better prediction performance with better indices. As for other metaheuristic algorithms(Crayfish, Reptile, and Redfox), the proposed SSA-XGBoost model in this study exhibits the best performance in terms of six indices (Best, Worst, Mean, Median, Std, Var). Moreover, Combined with the comparison results provided by the convergence diagram and box plots. In practice, the SSA-XGBoost model can be applied to monitor and forecast the population.
(3) In view of the fact that the dataset used in this study is characterized by tabular data, and that the improved versions of RNNs such as LSTM and GRU that we have selected do not perform as well as XGBoost in our experimental results, it confirms that XGBoost is inherently superior in dealing with tabular data relative to deep models such as RNNs. The SSA-XGBoost model yields a higher prediction accuracy and better evaluation indices. For the three evaluation indices of MSE, MAE, and R^2^, the SSA-XGBoost model can achieve more improvements, which effectively indicates the powerful prediction performance and high robustness of the SSA-XGBoost model and provides a new way of thinking about time series prediction research.

The proposed method in this paper can effectively forecast the population, which could expand in many specific cases such as optimizing public services by predicting demand, resource allocation in urban planning by forecasting population growth, or traffic management by anticipating congestion patterns.

Due to computational resource constraints, the study models are researched mainly based on the national census data and mobile information data, which is limited in geographical and temporal fineness. The model prediction is only yearly and the data management is limited to Hebei Province. Future research can incorporate sound data for a more comprehensive study.

## Supporting information

S1 Data Dataset. (ZIP)
